# Altered protein phosphatase 2A methylation and Tau phosphorylation in the young and aged brain of methylenetetrahydrofolate reductase (MTHFR) deficient mice

**DOI:** 10.3389/fnagi.2014.00214

**Published:** 2014-08-22

**Authors:** Jean-Marie Sontag, Brandi Wasek, Goce Taleski, Josephine Smith, Erland Arning, Estelle Sontag, Teodoro Bottiglieri

**Affiliations:** ^1^School of Biomedical Sciences and Pharmacy, Faculty of Health, University of Newcastle and Hunter Medical Research InstituteCallaghan, NSW, Australia; ^2^Institute of Metabolic Disease and Baylor Research Institute, Baylor University Medical CenterDallas, TX, USA

**Keywords:** Alzheimer’s disease, folate, LCMT1, MTHFR, methylation, PP2A, Tau phosphorylation

## Abstract

Common functional polymorphisms in the methylenetetrahydrofolate reductase (MTHFR) gene, a key enzyme in folate and homocysteine metabolism, influence risk for a variety of complex disorders, including developmental, vascular, and neurological diseases. MTHFR deficiency is associated with elevation of homocysteine levels and alterations in the methylation cycle. Here, using young and aged *Mthfr* knockout mouse models, we show that mild MTHFR deficiency can lead to brain-region specific impairment of the methylation of Ser/Thr protein phosphatase 2A (PP2A). Relative to wild-type controls, decreased expression levels of PP2A and leucine carboxyl methyltransferase (LCMT1) were primarily observed in the hippocampus and cerebellum, and to a lesser extent in the cortex of young null *Mthfr*^−/−^ and aged heterozygous *Mthfr*^+/−^ mice. A marked down regulation of LCMT1 correlated with the loss of PP2A/Bα holoenzymes. Dietary folate deficiency significantly decreased LCMT1, methylated PP2A and PP2A/Bα levels in all brain regions examined from aged *Mthfr*^+/+^ mice, and further exacerbated the regional effects of MTHFR deficiency in aged *Mthfr*^+/−^ mice. In turn, the down regulation of PP2A/Bα was associated with enhanced phosphorylation of Tau, a neuropathological hallmark of Alzheimer’s disease (AD). Our findings identify hypomethylation of PP2A enzymes, which are major CNS phosphatases, as a novel mechanism by which MTHFR deficiency and *Mthfr* gene-diet interactions could lead to disruption of neuronal homeostasis, and increase the risk for a variety of neuropsychiatric disorders, including age-related diseases like sporadic AD.

## Introduction

**Figure 1:**
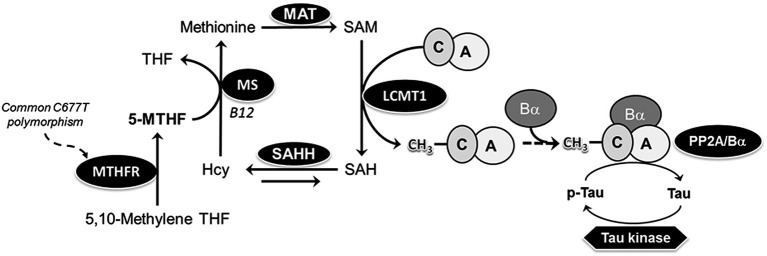
**Model of the link between MTHFR, folate, PP2A methylation and Tau phosphorylation**. 5-MTHF, produced via the action of MTHFR, is essential for remethylation of homocysteine (Hcy) to methionine, the precursor of S-adenosylmethionine (SAM), the universal methyl donor. Methylation of PP2A catalytic subunit on Leu-309 by the dedicated SAM-dependent LCMT1 methyltransferase promotes the biogenesis of PP2A/Bα heterotrimers, the primary Tau Ser/Thr phosphatases in the brain. Common C677→T polymorphisms in the *mthfr* gene induce mild MTHFR deficiency, leading to reduced 5-MTHF levels. Folate deficiency promotes accumulation of Hcy, which may be converted back to SAH, a potent inhibitor of LCMT1 activity. *MAT, methionine adenosine transferase; MS, methionine synthase; MTHF, methyltetrahydrofolate; SAHH, SAH hydrolase; THF, tetrahydrofolate*.

5,10-methylenetetrahydrofolate reductase (MTHFR) is the rate-limiting enzyme for converting folate to its active form, methylfolate (5-MTHF). 5-MTHF is a necessary co-factor for remethylation of homocysteine to methionine, which is essential for production of S-adenosylmethionine (SAM), the universal methyl donor (Figure 1). Severe MTHFR deficiency is the most common inborn error of folate metabolism, and leads to decreased red cell folate levels, elevated plasma total homocysteine (tHcy) levels, homocysteinuria, hypomethioninemia, and impaired cellular methylation potential (Goyette et al., 1996). It is associated with variable clinical outcomes, ranging from early neonatal demise to later-onset damage to the nervous and vascular systems, resulting in varying degrees of developmental delay, neurological impairment, motor dysfunction, gait abnormalities, seizures, and thrombotic events. Notably, there is a relatively high prevalence of functional genetic *Mthfr* polymorphisms in the general population (Botto and Yang, 2000). Among them, the common human *Mthfr* 677C→T gene polymorphism is associated with mild MTHFR deficiency, and is the most frequent cause of hyperhomocysteinemia (Leclerc and Rozen, 2007). *In vitro*, the C to T change at nucleotide position 677 leads to production of thermolabile MTHFR enzymes with ~35% and ~65% decreased MTHFR activity in heterozygous 677CT and homozygous 677TT individuals, respectively (Rozen, 1997). Of particular relevance to the CNS, the *Mthfr* 677CT polymorphism has been clinically identified as a risk factor for development neural tube defects (Botto and Yang, 2000), vascular disease and stroke (McNulty et al., 2012), and a variety of neuropsychiatric diseases, including Down syndrome (Hobbs et al., 2000), epilepsy (Wu et al., 2014), migraine (Liu et al., 2014), depression, schizophrenia and bipolar disorder (Gilbody et al., 2007). Homozygosity for *Mthfr* 677TT is also associated in selected populations with age-related neurodegenerative diseases such as Alzheimer’s disease (AD; Kageyama et al., 2008; Wang et al., 2008; Hua et al., 2011; Coppede et al., 2012) and Parkinson’s disease (Wu et al., 2013).

Despite numerous clinical studies, the effects of MTHFR deficiency at the molecular level are not well understood. By affecting folate and homocysteine metabolism, MTHFR polymorphisms have the potential to disrupt the transfer of one-carbon units, thereby influencing many methylation-sensitive targets (Figure 1). We hypothesized that one of them might be protein phosphatase 2A (PP2A), a large family of essential Ser/Thr protein phosphatases (Sontag and Sontag, 2014). Significantly, PP2A is regulated by Leucine carboxyl methyltransferase 1 (LCMT1)-dependent methylation of its catalytic C subunit (PP2Ac), which influences PP2A holoenzyme biogenesis and substrate specificity (De Baere et al., 1999; Leulliot et al., 2004; Stanevich et al., 2011; Tsai et al., 2011). We have reported *in vivo* that low folate status (Sontag et al., 2008) and hyperhomocysteinemia (Sontag et al., 2007), lead to down regulation of expression levels of LCMT1, PP2A methylation, and PP2A holoenzymes containing the regulatory Bα (or PPP2R2A) subunit (PP2A/Bα). This correlates with increased phosphorylation of specific PP2A/Bα substrates, such as Tau proteins (Sontag and Sontag, 2014). The accumulation of phosphorylated Tau (p-Tau) species, a neuropathological hallmark of AD and other tauopathies, mediates neurotoxicity in AD mouse models (Wang et al., 2013). Here, using young and aged *Mthfr* knockout mouse models, we investigated whether MTHFR deficiency and *Mthfr*-low folate gene-diet interactions can potentially affect the methylation state of PP2A, and its substrate, Tau.

## Materials and methods

### Materials

Unless indicated, all chemicals used in this study were obtained from Sigma-Aldrich (St. Louis, MO and Castle Hill, Australia).

### *Mthfr* mice and folate deficiency

All experiments with mice were performed in accordance with protocols approved by the Institutional Animal Care and Use Committee at Baylor Research Institute. Wild-type (Wt) *Mthfr*^+/+^, heterozygous (het) *Mthfr*^+/−^ and homozygous (null) *Mthfr*^−/−^ knockout mice in the C57Bl/6 background (Chen et al., 2001) were obtained from Dr. Rima Rozen (The Research Institute of the McGill University Health Center, Montreal, Quebec, Canada). Mice were bred, genotyped and housed in cages with a maximum of 4 mice per cage, maintained in a temperature-controlled animal facility on a 12-h light dark cycle, and were allowed access to food and water *ad libitum*. At ~5 weeks of age, a subset of mice (*n* = 10 female per genotype) fed a normal chow diet was sacrificed by CO_2_ asphyxiation. At ~16 months of age, subsets of wt mice were placed for 6 months on an amino acid defined diet (*n* = 10 female per group) with either a normal folate (6.7 mg/kg, NF) or low folate (0.2 mg/kg, LF) content. These custom diets (Harland Teklad) also contained succinylsulfathiazole (10 g/kg) to inhibit gastrointestinal bacterial growth and prevent absorption of folate from this source (Sontag et al., 2008). The diets did not induce any statistically significant changes in mouse weight in any of the group studied (mean weight in grams ± SD: Wt mice on NF diet, 37 ± 9 at baseline, 40 ± 7 at 22 months; het mice on NF diet, 43 ± 7 at baseline, 41 ± 6 at 22 months; wt mice on LF diet, 35 ± 7 g at baseline, 34 ± 3 g at 22 months; het mice on LF diet, 46 ± 14 g at baseline, 40 ± 10 g at 22 months). At ~22 months of age, mice were sacrificed by CO_2_ asphyxiation. Blood was obtained by cardiac puncture and brain tissue rapidly removed for regional dissection (Sontag et al., 2008). Tissues were stored at −80°C until time of analysis.

### Plasma metabolite analysis

Plasma tHcy was determined by high pressure liquid chromatography with fluorescence detection (Ubbink et al., 1991). Plasma 5-MTHF was measured by liquid chromatography mass spectrometry as previously described (Nelson et al., 2004).

### Determination of protein expression and PP2A methylation levels

Total brain homogenates (0.1 g tissue/ml of buffer) were prepared from each brain region exactly as described previously (Sontag et al., 2007, 2008). Aliquots were analyzed immediately for PP2A methylation or kept frozen at −80°C for future analyses. Equivalent aliquots (5 μl) of brain homogenates were resolved on 4–12% Bis-Tris gels using the NU-PAGE system (Thermo Fisher Scientific) followed by quantitative Western blotting. Precision Plus Protein™ Standards (BIO-RAD) were used as molecular weight standards. Antibodies against LCMT1 (clone 4A4, Merck Millipore #05-592), Bα (clone 2G9, Merck Millipore #05-849), total Tau (rabbit anti-Tau T-1308-1, rPeptide) and Tau phosphorylated at the PHF-1 epitope (Greenberg et al., 1992) were used to assess protein expression levels exactly as described previously (Sontag et al., 2007, 2008; Bottiglieri et al., 2012). Methylation of PP2A was determined using two methods. First, western blots of brain tissue samples were probed with monoclonal anti-methyl specific PP2A antibodies (clone 2A10, Merck Millipore #04-1479), followed by re-probing with methylation-insensitive antibodies (clone 46, BD Biosciences #610556) (Sontag et al., 2007). In the second method, equivalent aliquots of brain tissue homogenates were incubated for 30 min at 37°C in the absence or presence of 0.2 N sodium hydroxide (NaOH). Alkaline treatment results in complete demethylation of PP2Ac at Leu-309. NaOH-treated and untreated samples were then analyzed by NU-PAGE electrophoresis followed by Western blotting with monoclonal anti-demethylated PP2Ac antibodies (clone 1D6, Merck Millipore #05-421) to detect demethylated (NaOH-untreated samples) and total PP2Ac (NaOH-treated samples) (Bottiglieri et al., 2012). All the blots were re-blotted with monoclonal anti-actin antibodies (clone 4, MAB1501 Merck Millipore) to allow normalization for protein loading. Western blotting was performed using Infrared IRDye^®^-labeled secondary antibodies and the Odyssey^TM^ Infrared imaging system (LI-COR Biosciences). Band intensity was determined using the associated Image Studio Lite version 3.1 Software (LI-COR Biosciences) to accurately quantify protein levels. Some samples were also analyzed in duplicate by Western blotting using chemiluminescence detection system (Pierce), followed by densitometry (Sontag et al., 2007).

### Statistics

Data were analyzed using one-way ANOVA with *post hoc* Tukey’s multiple comparison test. Differences with *p*-values < 0.05 were considered statistically significant.

## Results

### MTHFR deficiency in young mice impairs brain LCMT1 protein expression and PP2A methylation in a region-specific manner

**Figure 2:**
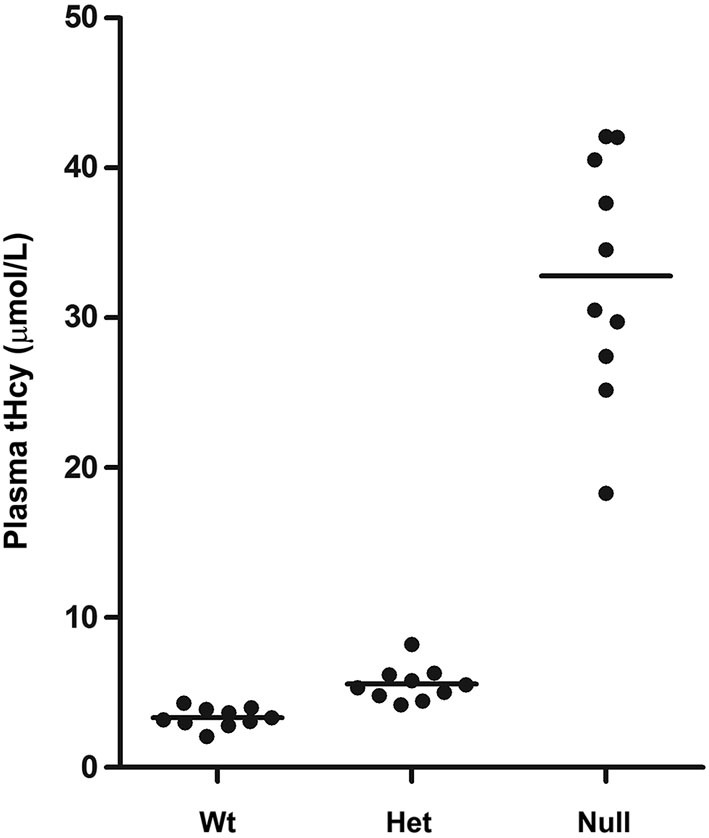
**Plasma tHcy levels in *Mthfr* knockout mice**. Plasma tHcy levels were measured in 5-week-old wt *mthfr*^+/+^, het *mthfr*^+/−^ and null *Mthfr*^−/−^ mice (*n* = 10 mice/ group).

**Figure 3:**
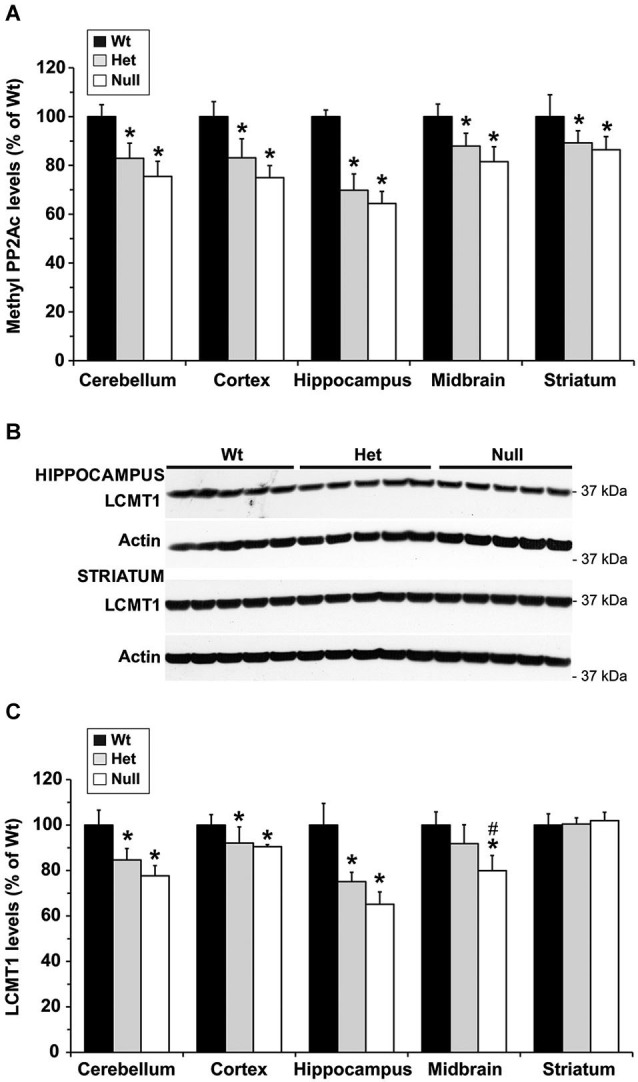
**Alterations in regional brain levels of methylated PP2Ac and LCMT1 in young mice models of mild and severe MTHFR deficiency**. Regional brain homogenates from 5-week-old wt *mthfr^+/+^* (black bars), het *mthfr*^+/−^ (gray bars), and null *Mthfr*^−/−^ (white bars) mice were analyzed by quantitative Western blotting for relative protein expression levels of methylated PP2Ac **(A)** and LCMT1 **(B,C)**. Values represent mean ± SD for 6 mice per group; * *p* < 0.05, het or null compared to wt mice; ^#^ *p* < 0.05, null vs. het mice. Representative LCMT1 blots of two brain regions are shown in **(B)**.

To investigate whether MTHFR deficiency can influence the methylation of PP2A and its substrate Tau, we first analyzed young murine models of mild (*Mthfr^+/−^*) and severe (*Mthfr*^−/−^) MTHFR deficiency. These well-characterized knockout mice have been shown to reproduce the biochemical and clinical consequences of *Mthfr* 677C→T polymorphisms in human (Chen et al., 2001; Ghandour et al., 2004). Relative to their 5-week-old wt littermates, there was an average ~1.6-fold increase in plasma tHcy levels in het *mthfr*^+/−^ mice, while null *Mthfr*^−/−^ mice exhibited a ~10-fold increase in plasma tHcy levels (Figure 2), in agreement with earlier studies (Chen et al., 2001). It is well established that impairment of MTHFR activity can lead to reduced 5-MTHF levels and elevated tHcy levels that compromise SAM-dependent methylation reactions (Chen et al., 2001). Significantly, the activity of LCMT1, the sole PP2A methyltransferase (MacKay et al., 2013), is dependent on SAM supply (Lee et al., 1996; Leulliot et al., 2004; Sontag et al., 2007; Tsai et al., 2011). Accordingly, we found that, relative to wt mice, methylation of PP2Ac was reduced in all the brain regions examined from het or null *mthfr* knockouts (Figure 3A). Of note, the extent of decrease in methylated PP2Ac levels was variable among regions, being highest in the hippocampus, and lowest in the striatum. We have previously reported that, besides affecting SAM-dependent LCMT1 methyltransferase activity, *in vivo* alterations in plasma folate and/or tHcy levels can also result in down regulation of brain LCMT1 at the protein level, through a yet unresolved mechanism (Sontag et al., 2007, 2008; Bottiglieri et al., 2012). Interestingly, a marked decrease in LCMT1 expression levels was observed in the hippocampus and cerebellum, and to a lesser extent in the cortex, but not in the midbrain and striatum from young het *Mthfr*^+/−^ mice, relative to wt mice (Figures 3B,C). Compared to their wt littermates, lower LCMT1 protein amounts were observed in all brain regions of null *Mthfr*^−/−^ mice, with the exception of the striatum. Notably, the down-regulation of LCMT1 expression was most prevalent in the hippocampus, wherein the loss of methylated PP2Ac was maximal (Figure 3A), in line with the observation that knock down of LCMT1 results in a significant reduction in PP2Ac methylation (Lee and Pallas, 2007; Sontag et al., 2008; MacKay et al., 2013). Together, these results suggest that MTHFR deficiency affect LCMT1 and PP2A methylation in a brain region-specific manner.

### Severe MTHFR deficiency in young mice induces a down regulation of PP2A/Bα and concomitant enhancement of p-Tau levels in the hippocampus and cerebellum, and to a lesser extent, in the cortex

**Figure 4:**
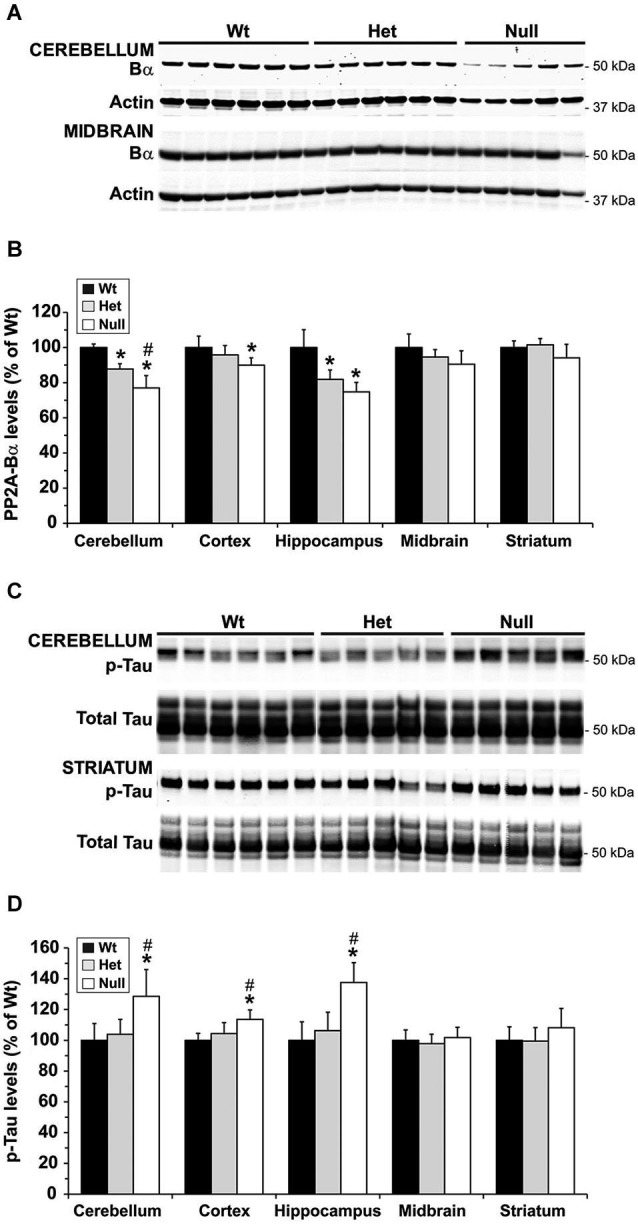
**Regional alterations in PP2A/Bα and p-Tau levels in young mice models of mild and severe MTHFR deficiency**. Regional brain homogenates from the same mice described in Figure 3 were comparatively analyzed by quantitative Western blotting for expression levels of PP2A/Bα **(A,B)** and Tau phosphorylated at the PHF-1 epitope **(C,D)**. Bar shading and symbols as in Figure 3. Representative blots of two regions are shown in **(A)** and **(C)**.

We and others have shown that, by reducing the formation of PP2A/Bα heterotrimers, down regulation of LCMT1 is accompanied with a preferential loss of endogenous PP2A/Bα isoforms (Lee and Pallas, 2007; Sontag et al., 2008). Likewise, the region-specific decrease in LCMT1 expression levels (Figure 3C) correlated with a similar reduction in PP2A/Bα expression levels (Figures 4A,B) in het and null *Mthfr* mice. As observed with LCMT1, the down regulation of PP2A/Bα expression levels was more prevalent in the hippocampal and cerebellar regions. Since the PP2A/Bα heterotrimer is a major Tau phosphatase (Sontag et al., 1996; Xu et al., 2008), we next investigated whether the reduction in PP2A/Bα amounts correlated with enhanced phosphorylation of endogenous Tau at the AD-like p-Ser396/Ser404 PHF-1 epitope. Levels of p-Tau (PHF-1) in each brain region were similar in wt and het MTHFR mice (Figures 4C,D). However, relative to control wt mice, a ~30–50% enhancement of Tau phosphorylation was observed in the hippocampus and cerebellum of null mice, the only regions wherein PP2A/Bα levels were markedly decreased (Figure 4B). A minor increase in p-Tau levels, which correlated with a smaller loss of PP2A/Bα (Figure 4B) was also observed in cortical homogenates of null mice. In contrast, relative to controls, there was no change in p-Tau or PP2A/Bα amounts measured in the midbrain and striatum. Together, these data point to an inverse relationship between PP2A/Bα and p-Tau levels. They also suggest that a certain threshold of LCMT1 and PP2A/Bα down regulation, which selectively occurs in the hippocampal, cerebellar and cortical regions of young null mice, is required to achieve a significant increase in Tau phosphorylation.

### Aging worsens the effects of mild MTHFR deficiency on p-Tau in susceptible regions

**Figure 5:**
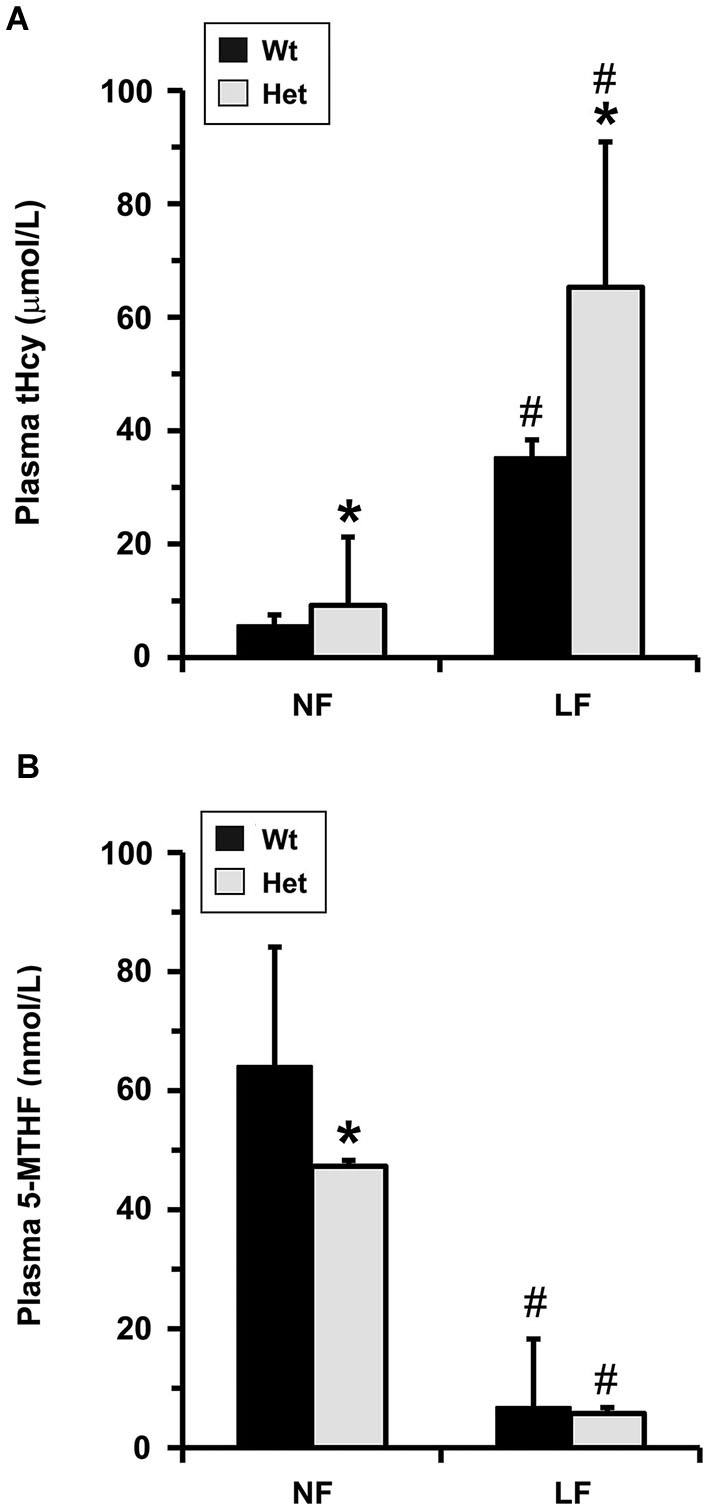
**Plasma metabolite levels in old mice models of mild MTHFR deficiency**. Plasma tHcy **(A)** and 5-MTHF **(B)** levels were determined in 22-month-old wt *mthfr^+/+^* and het *mthfr*^+/−^ mice that had been fed for 6 months an amino acid defined diet with either a normal folate (NF) or a low folate (LF) content. Values represent mean ± SD for 10 mice per group; * *p* < 0.05, het vs. wt in the same diet group; ^#^ *p* < 0.05, LF vs. NF in the same genotype.

**Figure 6:**
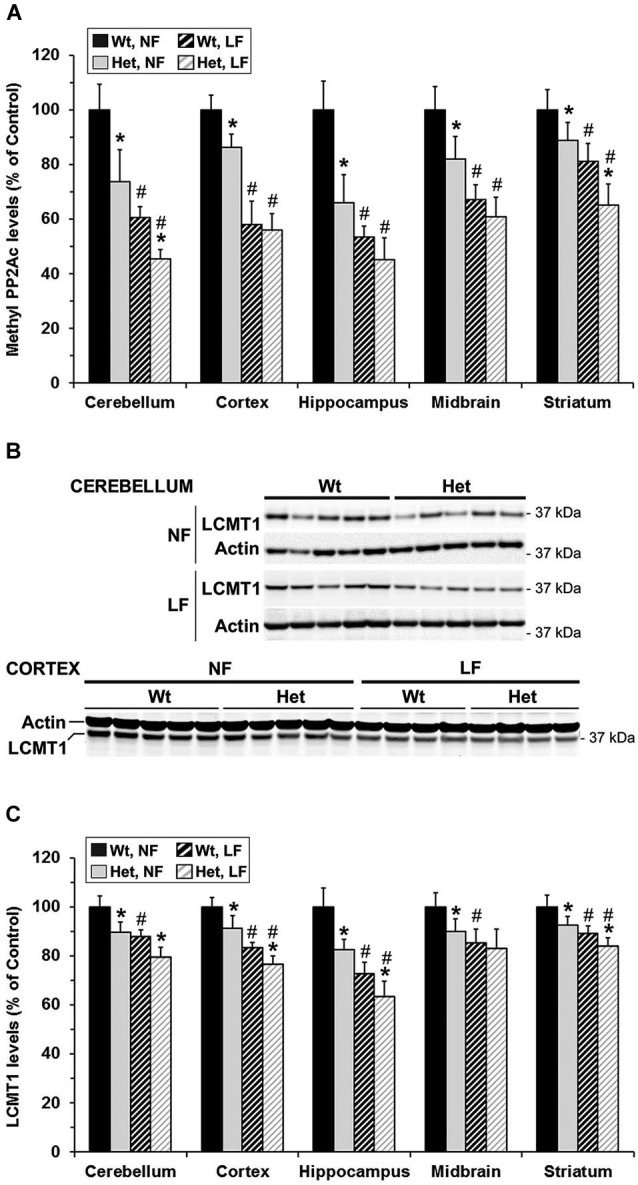
**Brain region-specific effects of folate and mild MTHFR deficiencies on PP2A methylation and LCMT1 expression levels**. 22-month-old wt *mthfr^+/+^* and het *mthfr*^+/−^ mice were fed for 6 months either a NF diet (Black bars, wt mice; gray bars, het mice) or a LF diet (hatched black bars, wt mice; hatched light gray bars, het mice). Relative methylated PP2Ac **(A)** and LCMT1 **(B,C)** protein expression levels were determined after quantitative Western blotting. Values represent mean ± SD for 9 mice per group; * *p* < 0.05, het vs. wt in the same diet group; ^#^ *p* < 0.05, LF vs. NF in the same genotype. Representative LCMT1 blots for two regions are shown in **(B)**.

**Figure 7:**
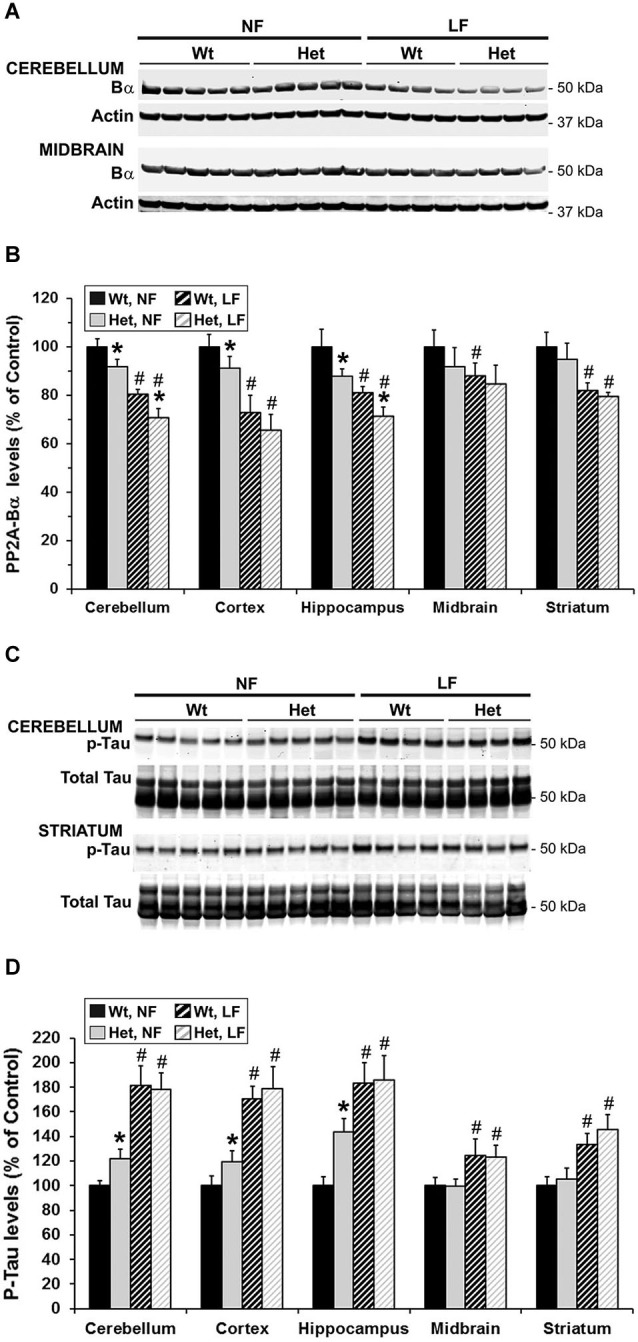
**Brain region-specific effects of folate and mild MTHFR deficiencies on PP2A/Bα and p-Tau expression levels**. Regional brain homogenates from the same mice described in Figure 6 were comparatively analyzed by quantitative Western blotting for relative protein expression levels of PP2A/Bα **(A,B)** and Tau phosphorylated at the PHF-1 epitope **(C–D)**. Bar shading and symbols as in Figure 6. Representative blots for two regions are shown in **(A)** and **(C)**.

Notably, *Mthfr* polymorphisms have been identified as a risk factor for AD, an age-related disorder (Wang et al., 2005, 2008; Kageyama et al., 2008; Hua et al., 2011; Coppede et al., 2012; Mansouri et al., 2013). Thus, we next addressed the hypothesis that normal aging could aggravate the detrimental effects of MTHFR deficiency on LCMT1, PP2A methylation and p-Tau. To that end, we comparatively analyzed regional brain homogenates from 22-month-old wt and het *Mthfr* mice fed an amino acid defined diet with normal folate content (normal folate diet, NF). Null *Mthfr*^−/−^ mice could not be used in these studies, since mice with severe MTHFR deficiency die prematurely as a result of atherosclerosis and other complications (Chen et al., 2001; Lawrance et al., 2011). Similar to the effects of MTHFR deficiency in young *Mthfr*^+/−^ mice (Figure 2), plasma tHcy levels were increased by an average of ~1.6-fold in old *Mthfr*^+/−^ mice, relative to wt controls (Figure 5A). Besides promoting hyperhomocysteinemia, MTHFR deficiency leads to altered folate distribution and reduction in plasma and brain 5-MTHF levels (Chen et al., 2001; Ghandour et al., 2004). Accordingly, plasma 5-MTHF levels were significantly decreased in old het vs. wt animals (Figure 5B). These metabolic changes were associated with a marked decrease of PP2Ac methylation in all the brain regions examined from het mice (Figure 6A), reinforcing the hypothesis that alterations in plasma tHcy and 5-MTHF levels can lead to inhibition of SAM-dependent, LCMT1-mediated PP2A methylation in the brain. Relative to controls, there was also a small but statistically significant down regulation of LCMT1 protein expression levels in all brain regions examined from old het MTHFR^+/−^ mice (Figures 6B,C); again, the loss of LCMT1 and methylated PP2Ac was more pronounced in the hippocampus. As observed in young *Mthfr*^+/−^ het mice, reduced PP2A/Bα levels (Figures 7A,B) correlated with enhanced p-Tau phosphorylation at the PHF-1 epitope (Figures 7C,D) in the hippocampus, cerebellum and cortex from old het *Mthfr*^+/−^ mice.

Overall, our data indicate that the MTHFR genotype very similarly influenced LCMT1, methylated PP2Ac and PP2A/Bα protein expression levels in regional brain homogenates from young and old het MTHFR^+/−^ mice, relative to their age-matched wt littermates. However, aging appeared to slightly worsen the effects of mild MTHFR deficiency on p-Tau, which selectively accumulated in the hippocampus, cerebellum and cortex of old, but not young het *Mthfr^+/−^* mice.

### Mild MTHFR deficiency worsens the effects of folate deficiency on LCMT1 and PP2A in susceptible brain regions

Interestingly, the relatively mild functional clinical consequences of *Mthfr* 677C→T polymorphisms are aggravated by deficiencies in dietary folate intake (Botto and Yang, 2000; Rozen, 2000; Schwahn and Rozen, 2001; Leclerc and Rozen, 2007). By functionally affecting the activity of methionine synthase, a key enzyme of the folate and methylation cycle (Figure 1), folate deficiency restricts Hcy metabolism, resulting in elevation of plasma tHcy levels (Fowler, 2005). Of particular significance, low folate status and elevated tHcy levels are independent risks factors for AD (Zhuo et al., 2011; Morris, 2012), and patients with AD have lower plasma folate levels (Lopes da Silva et al., 2014). Based on these critical observations, we examined whether dietary folate deficiency can affect the levels of LCMT1, PP2A and p-Tau levels in old mice, either by itself or in synergy with mild MTHFR deficiency. Low folate status was induced by feeding ~16-month-old mice an amino acid defined diet with reduced folate content (low folate diet, LF) for 6 months. As predicted, plasma tHcy levels were significantly increased (Figure 5A), and 5-MTHF levels were dramatically reduced (Figure 5B) in 22-month-old wt mice fed the LF diet, compared to age-matched control animals receiving the NF diet. Mild MTHFR deficiency in het *Mthfr*^+/−^ mice aggravated the hyperhomocysteinenia induced by the LF diet (Figure 5A, het vs. wt mice). However, it failed to further affect plasma 5-MTHF levels (Figure 5B, het vs. wt mice), probably because those were already maximally decreased in response to the prolonged dietary folate deficiency. Comparative Western blot analyses of tissue homogenates from wt mice fed a NF or LF diets showed that folate deficiency alone led to a significant down regulation of methylated PP2Ac (Figure 6A), LCMT1 (Figures 6B,C) and PP2A/Bα (Figures 7A,B), and concomitant accumulation of p-Tau (Figures 7C,D) in all brain regions examined. Again, the LF diet-induced enhancement of Tau phosphorylation was especially prominent in the hippocampus, cerebellum and cortex. Comparative analysis of het mice showed that mild MTHFR deficiency further exacerbated the effects of folate deficiency on PP2Ac methylation (Figure 6A), LCMT1 (Figure 6C) and PP2A/Bα expression levels (Figure 7B) in a brain region-specific manner. Despite inducing a greater reduction in the amounts of PP2A/Bα levels in susceptible regions (hippocampus, cerebellum), there was no apparent effect of the *Mthfr*^+/−^ genotype on p-Tau levels in mice fed the LF diet (Figures 7C,D). This suggests that endogenous Tau phosphorylation had already peaked to its maximum level in response to the dietary folate deficiency alone.

## Discussion

### Severe MTHFR deficiency affects LCMT1-dependent PP2A methylation, PP2A/Bα and p-Tau expression levels in the hippocampus, cerebellum and cortex

It is well established that disturbances in folate metabolism exert a detrimental effect on the brain by affecting the supply of methyl groups that are critical for normal homeostasis; indeed, individuals with severe MTHFR deficiency have a variety of neurological problems and decreased mental abilities (Watkins and Rosenblatt, 2012). Null *Mthfr^−/−^* mice have been found to have abnormalities in the size and/or structure of the cerebellum, cortex and hippocampus, and to exhibit memory impairment and other behavioral anomalies reminiscent of those encountered in patients with severe MTHFR deficiency (Chen et al., 2001, 2005; Jadavji et al., 2012). Here, using *Mthfr*^−/−^ knockout mice, we show for the first time that severe MTHFR deficiency can impair LCMT1-dependent PP2A methylation. PP2A is a family of abundant brain Ser/Thr phosphatases that collectively participate in nearly all aspects of neuronal homeostasis. Of particular interest, biogenesis of major PP2A/Bα isoforms that are primary brain enzymes that dephosphorylate Tau, is critically influenced by LCMT1-mediated PP2Ac methylation (Lee and Pallas, 2007; Sontag et al., 2008). Decreased PP2A methylation and PP2A/Bα expression levels correlate with enhanced tau phosphorylation in several mouse models of altered one-carbon metabolism Reviewed in Sontag and Sontag (2014) and in diabetic mice (Papon et al., 2013). Notably, altered phosphorylation of Tau is a central pathological event believed to initiate Tau aggregation and dysfunction, ultimately resulting in neurodegeneration and cognitive decline in AD and other tauopathies. There is also substantial evidence that the accumulation of p-Tau is a central mediator of amyloid-β toxicity and synaptic deficits in AD (Liao et al., 2014). Significantly, we demonstrate *in vivo* that severe MTHFR deficiency is associated with down regulation of LCMT1 and PP2A/Bα, and concomitant enhancement of Tau phosphorylated at the AD-like PHF-1 epitope in the hippocampus and cerebellum, and to a lesser extent in the cortex. Alterations in p-Tau in the hippocampus, a region critically involved in spatial learning and memory, could contribute to some of the cognitive deficits previously reported in *Mthfr*^−/−^ mice (Chen et al., 2001, 2005; Jadavji et al., 2012). Because of the prominent position of PP2A in neuronal signaling, deregulation of LCMT1-dependent PP2A methylation also provides a novel insight into the mechanisms by which MTHFR deficiency can negatively impact neuronal function in specific susceptible regions (hippocampus, cerebellum, cortex) identified in earlier *Mthfr*^−/−^ mouse studies (Chen et al., 2001, 2005; Jadavji et al., 2012). Interestingly, the hippocampus and cortex are especially vulnerable to AD-related pathological p-Tau changes, while the cerebellum is traditionally spared in this neurodegenerative disorder. Accordingly, using a large series of neuropathologically confirmed autopsy cases of AD, we have shown that down regulation of LCMT1, PP2Ac methylation (Sontag et al., 2004a) and PP2A/Bα (Sontag et al., 2004b) occurs in AD-affected cortical and hippocampal regions, where it correlates with the accumulation of p-Tau. In contrast, we have not observed a concomitant loss of these enzymes in the AD cerebellum, a region wherein neuronal p-Tau lesions do not accumulate. Yet, down regulation of PP2A methylation in wt mice increases cerebellar p-Tau levels (Bottiglieri et al., 2012) and excitoxicity influences PP2A-dependent tau phosphorylation in cerebellar granule cells (Kuszczyk et al., 2009), further demonstrating the tau regulatory function of PP2A in the cerebellum. While MTHFR deficiency-induced cerebellar p-Tau changes may not directly relate to the neurodegenerative process in AD, it is worth mentioning that low concentrations of 5-MTHF in the cerebrospinal fluid have been linked to cerebellar atrophy and neurological disorders in children (Grapp et al., 2012).

We noticed that severe MTHFR deficiency affected PP2Ac methylation in all brain regions, albeit with distinct intensity. Yet, this did not automatically translate into the loss of PP2A/Bα, which better correlated with the down regulation of LCMT1. These data suggest the existence of additional brain-region specific regulation of these enzymes- for instance transcription, synthesis, degradation- and/or compensatory mechanisms that will need to be investigated in future studies. Alternatively, a certain threshold loss of LCMT1 activity/expression and PP2Ac methylation may need to be reached to observe an effect on PP2A/Bα expression levels. Inherent limitations of quantitative Western blotting may preclude the measurement of subtle changes in the expression of these PP2A isoforms. Moreover, while both down regulation of SAM-dependent LCMT1 activity and LCMT1 protein expression levels can inhibit PP2Ac methylation (Sontag et al., 2008), alterations in the protein amounts of PME-1, the dedicated PP2A methylesterase, could also contribute to the observed accumulation of demethylated PP2Ac. For instance, previous studies have shown that incubation of primary neurons with folate antagonists can induce an up-regulation of PME-1 that correlates with an increase of endogenous demethylated PP2A (Yoon et al., 2007). We have also observed changes in PME-1 levels in wt mice fed for 2 months on a folate deficient diet (Sontag et al., 2008). Unfortunately, due to shortage of tissue material, we were unable in the present study to measure expression levels of PME-1 in corresponding brain regions of our *mthfr* knockouts.

### Mild MTHFR deficiency affects LCMT1, PP2A methylation, PP2A/Bα and p-Tau (PHF-1) in a brain-region specific manner

There is substantial evidence that common single human *Mthfr* polymorphisms that have a frequency of up to 50% in certain populations (Zappacosta et al., 2014), modify the risk for numerous diseases (Nazki et al., 2014). Of particular interest here, *Mthfr* polymorphisms that result in mild MTHFR deficiency have been identified as a risk factor for AD in selected populations (Wang et al., 2005, 2008; Kageyama et al., 2008; Hua et al., 2011; Coppede et al., 2012; Mansouri et al., 2013). Moreover, epigenetic mechanisms involving *Mthfr* and altered methylation homeostasis may predispose to development of late-onset AD (Wang et al., 2008). Interestingly, we found that mild MTHFR deficiency was able to induce a variable down regulation of PP2Ac methylation in all brain regions examined, and the intensity of these effects was comparable in young or old *Mthfr*^+/−^ mice, relative to age-matched wt littermates. A similar small loss in LCMT1 protein levels was observed in the hippocampal, cerebellar and cortical regions of young and old *Mthfr* knockouts. In 22-month-old *Mthfr*^+/−^ mice, reduced expression of PP2A/Bα again correlated with increased levels of p-Tau, in agreement with the role of this isoform as a primary Tau phosphatase. In contrast, we were unable to detect an increase in Tau phosphorylation at the PHF-1 epitope in young *Mthfr*^+/−^ animals, relative to controls, despite the small decrease in PP2A/Bα levels observed in the hippocampus and cerebellum. It is possible that Tau protein Ser/Thr kinases could compensate for the small regional decrease of PP2A/Bα and maintain a steady state of Tau phosphorylation in the brain of 5-week-old mice. Those compensatory mechanisms might become compromised with aging, or the small loss of PP2A/Bα could have a compounding effect with time, resulting in the increased tau phosphorylation observed in older *Mthfr*^+/−^ mice. Another alternative is that Tau could become phosphorylated at other epitopes not studied here, due to the lack of sufficient amounts of regional brain tissue. The PHF-1 is considered a late phospho-Tau epitope in AD, and PP2A methylation regulates many other “earlier” Tau phosphorylation sites (Sontag et al., 2007) that could be potentially sensitive to the effects of mild MTHFR deficiency in young animals. However, we were unable to detect increased phosphorylation of Tau at pSer422 in cortical homogenates from young het *Mthfr* mice (data not shown).

### Interactions between the *Mthfr* genotype and dietary folate deficiency differentially affect LCMT1, PP2A and p-Tau in distinct brain regions

We have previously reported that LCMT1, PP2Ac methylation and PP2A/Bα become down regulated, and Tau phosphorylation is increased in the cerebellum and cortex, and to a lesser extent in the midbrain and striatum of 4-week-old wt mice fed for 2 months a LF diet (Sontag et al., 2008). Here, we found similar results in 22-month-old wt mice that had been fed for 6 months a LF diet (Figures 6, 7), except that the prolonged diet induced more intense effects. We also show here that the hippocampus is especially sensitive to the effects of dietary folate deficiency, which resulted in nearly 30% loss of LCMT1, 50% loss of methylated PP2Ac, and concomitant doubling of p-Tau (PHF-1) levels in wt mice fed the LF diet, relative to control animals fed the NF diet. Whether down regulation of LCMT1 results either from prolonged inhibition of its methyltransferase activity -due to hyperhomocysteinemia and decreased cellular methylation potential- or other regulatory mechanisms remains to be defined. Of note, folate deficiency aggravated the negative effects of mild MTHFR deficiency on LCMT1 and PP2A methylation in a region-specific manner. Dietary folate deficiency also synergized with the *Mthfr^+/−^* genotype to further exacerbate the loss of PP2A/Bα in the hippocampus and cerebellum. However, this did not lead to a further enhancement of p-Tau levels in these regions, probably because p-Tau was already maximally increased in response to the LF diet alone. Altogether, these findings suggest brain-region specific responses to the effects of both dietary folate and MTHFR deficiencies. Further studies will be required to determine whether those are related to either differential expression patterns of all proteins involved, or compensatory mechanisms. Nevertheless, our findings clearly establish deregulated LCMT1 and PP2A as novel brain intermediates of the detrimental effects of MTHFR deficiency. Our results also unveil for the first time a link between MTHFR deficiency and deregulation of Tau phosphorylation, which could explain -at least in part- why epidemiological studies have identified *Mthfr* polymorphisms as risk factors for AD. Our experimental data also reinforce the notion that this risk may be modulated by folate intake. Significantly, the age-related decline of brain 5-MTHF levels (Bottiglieri et al., 2000) is associated with increased levels of p-Tau and cognitive decline (Herrmann and Obeid, 2007). Besides promoting tau phosphorylation, down regulation of LCMT1 and PP2A methylation can also affect amyloid protein precursor phosphorylation and processing (Sontag et al., 2007). Whether MTHFR polymorphisms also influence AD risk by altering amyloidogenesis remains to be investigated.

Due to the critical and ubiquitous cellular functions of PP2A enzymes, MTHFR-mediated PP2A dysfunction is likely to have functional consequences reaching far beyond the scope of Tau deregulation and AD. For instance, *Mthfr* polymorphisms influence the risk for many other neurological, vascular and developmental disorders that affect the CNS, as well as the pharmacodynamics of antifolates and effectiveness and toxicity of many therapeutic drugs (Schwahn and Rozen, 2001).

## Author contributions

Jean-Marie Sontag, Estelle Sontag and Teodoro Bottiglieri participated in the design and conceptualization of the study, analysis and interpretation of data, and editing of the manuscript for intellectual content.

Estelle Sontag wrote the manuscript; Jean-Marie Sontag, Goce Taleski and Josephine Smith participated in the acquisition and interpretation of Western blot data; Brandi Wasek did all the experimental mouse work; Erland Arning and Brandi Wasek performed metabolite analyses.

## Conflict of interest statement

Teodoro Bottiglieri reports having been the chairman of the Advisory Board for Methylation Sciences Inc., holding stock options in Methylation Sciences Inc., Scientific consultant to Pamlab LLC, and having received research funding from Pamlab LLC.
